# The cross-talk of HIV-1 Tat and methamphetamine in HIV-associated neurocognitive disorders

**DOI:** 10.3389/fmicb.2015.01164

**Published:** 2015-10-23

**Authors:** Sonia Mediouni, Maria Cecilia Garibaldi Marcondes, Courtney Miller, Jay P. McLaughlin, Susana T. Valente

**Affiliations:** ^1^Department of Infectious Diseases, The Scripps Research Institute, Jupiter, FL, USA; ^2^Department of Molecular and Cellular Neurosciences, The Scripps Research Institute, La Jolla, CA, USA; ^3^Department of Metabolism and Aging, The Scripps Research Institute, Jupiter, FL, USA; ^4^Department of Neuroscience, The Scripps Research Institute, Jupiter, FL, USA; ^5^Department of Pharmacodynamics, University of Florida, Gainesville, FL, USA

**Keywords:** methamphetamine, HAND, HIV-1 Tat, antiretroviral therapy, neurotoxicity

## Abstract

Antiretroviral therapy has dramatically improved the lives of human immunodeficiency virus 1 (HIV-1) infected individuals. Nonetheless, HIV-associated neurocognitive disorders (HAND), which range from undetectable neurocognitive impairments to severe dementia, still affect approximately 50% of the infected population, hampering their quality of life. The persistence of HAND is promoted by several factors, including longer life expectancies, the residual levels of virus in the central nervous system (CNS) and the continued presence of HIV-1 regulatory proteins such as the transactivator of transcription (Tat) in the brain. Tat is a secreted viral protein that crosses the blood–brain barrier into the CNS, where it has the ability to directly act on neurons and non-neuronal cells alike. These actions result in the release of soluble factors involved in inflammation, oxidative stress and excitotoxicity, ultimately resulting in neuronal damage. The percentage of methamphetamine (MA) abusers is high among the HIV-1-positive population compared to the general population. On the other hand, MA abuse is correlated with increased viral replication, enhanced Tat-mediated neurotoxicity and neurocognitive impairments. Although several strategies have been investigated to reduce HAND and MA use, no clinically approved treatment is currently available. Here, we review the latest findings of the effects of Tat and MA in HAND and discuss a few promising potential therapeutic developments.

## Introduction

The human immunodeficiency virus 1 (HIV-1) affects 35.3 million individuals worldwide (UNAIDS). The entry of the virus in the central nervous system (CNS) early during infection, and the relatively low penetration of current antiretroviral therapies (ART) in the brain, results in neurological complications collectively called HIV-associated neurocognitive disorders (HAND; Davis et al., 1992; reviewed in Gray et al., 1993; reviewed in Owen and Khoo, 2004; reviewed in Miller et al., 2008; Valcour et al., 2012; reviewed in Spudich, 2013). HAND can be subdivided into asymptomatic neurocognitive impairment (ANI), mild neurocognitive disorder (MND), and severe forms of HIV-associated encephalitis/dementia (HAD). Patients affected with ANI and MND score poorly in neuropsychological testing, but their everyday functioning is only minimally impacted. In contrast, HAD patients experience severe neurological failure that severely hampers their daily lives. Characteristic HAD symptoms include inflammation of the brain, decline in cognitive functions (memory, concentration, attention, and executive control), reduced motor function (loss of dexterity and coordination) and the mimicking of other dementia symptoms, such as in Alzheimer’s and Parkinson’s diseases. In addition, HAD patients present comorbid behavioral changes such as unipolar depression (apathy and loss of motivation), fatigue and social withdrawal (reviewed in Antinori et al., 2007). ART has considerably decreased the early occurrence of HAD, but the diagnosis of moderate neurocognitive impairments continues to increase (reviewed in Cisneros and Ghorpade, 2012). This increase is primarily attributed to longer life spans, the long-term toxicity of ART, and the development of resistance to therapy (reviewed in Pardo et al., 2001; reviewed in Lv et al., 2015; reviewed in Underwood et al., 2015).

The best accepted model to explain how HIV-1 gains access to the CNS is the “Trojan horse” hypothesis, in which infected monocytes and macrophages cross the blood–brain barrier (BBB) and subsequently infect non-neuronal cells including perivascular macrophages and glial cells (reviewed in Haase, 1986). Macrophages and microglia are the main supporters of viral replication in the brain (reviewed in Bagashev and Sawaya, 2013), whereas astrocytes are susceptible to HIV-1 infection but alone are not thought to support productive infection (reviewed in Bagashev and Sawaya, 2013). Pericytes have also been suggested as cells that may become infected by HIV-1, although at low levels, and be modified as a result of the presence of HIV-1 in the brain, thereby further contributing to BBB disruption (Nakagawa et al., 2012). Even though HIV-1 does not infect neurons, a major aspect of HAND is neuronal damage and apoptosis (reviewed in Kovalevich and Langford, 2012). Two mechanistic models of toxicity have been proposed: direct and indirect (or “bystander”; reviewed in Lindl et al., 2010). The direct model suggests that viral proteins released from infected cells cause neuronal death through a direct interaction with neurons. The indirect model proposes the death of neurons through the activity of soluble factors released as a result of inflammatory responses to viral particles or changes in the brain environment by non-neuronal cells. Regardless of the means, among the consequences of HIV infection is synaptodendritic injury to neurons that closely correlates to the occurrence and severity of neurocognitive disorders (reviewed in Ellis et al., 2007).

This synaptodendritic injury may be driven by viral replication in non-neuronal cells and is defined by structural and chemical changes occurring at neuronal synapses, resulting in a disturbance of communication between neurons responsible for behaviors such as memory, learning, and executive functions (reviewed in Ellis et al., 2007; reviewed in Nath and Steiner, 2014). Experimental studies in mice, monkeys or in human brain tissues confirm that exposure to HIV and the transactivator of transcription (Tat) protein induce alterations in neuronal morphology (reviewed in Ellis et al., 2007; Bertrand et al., 2014; reviewed in Nath and Steiner, 2014). These morphological changes associated with synaptodendritic injury may involve mitochondrial dysfunction (Greenwood et al., 2007). Notably, synaptodendritic injury may be reversible depending on the degree of injury (an example of “brain plasticity”; reviewed in Ellis et al., 2007). Accordingly, the addition of neuroprotective and regenerative factors to ART has been proposed to reduce HAND progression (reviewed in Ellis et al., 2007).

Among the viral proteins that play a role in neuropathogenesis, Tat, is a significant contributor. Although the main function of Tat is to regulate HIV-1 transcription, it has also been implicated in the dysregulation of inflammatory processes, induction of oxidative stress, excessive activation of neurotransmitters, modulation of apoptotic pathways, and irregular neurogenesis (see “Tat Neurotoxicity”, below). Indeed, elevated levels of Tat mRNA are commonly found in post-mortem brain samples from individuals with HAD (Wesselingh et al., 1993; Wiley et al., 1996; Hudson et al., 2000).

Beyond this, drug abuse correlates with an increased risk of exposure to HIV-1. The world drug report of 2014 (UNAIDS) describes that the prevalence of HIV infection among injecting drug users is 22–50 times higher than in the general population, depending on the country. The abuse of drugs largely prevalent in the US and worldwide, particularly the strong psychostimulant methamphetamine (MA), is correlated with increased neurotoxicity and immune system impairments, with a consequential exacerbation of HAND (Potula and Persidsky, 2008). It has been demonstrated in human and animal studies that the neurotoxicity caused by MA and Tat (or HIV-1 infection), results in greater cognitive and locomotor impairments than with either MA or Tat (or HIV-1) alone (see “Tat and Methamphetamine Neurotoxicity”).

This review will focus on the effects of Tat, MA, and their concerted effects in the CNS. Given that no treatment is yet available against the neurotoxic activity of Tat (Mediouni et al., 2012), or the additive detrimental effects of both Tat and MA, we will conclude with a discussion of some promising candidates emerging in the literature.

## Tat Neurotoxicity

**FIGURE 1:**
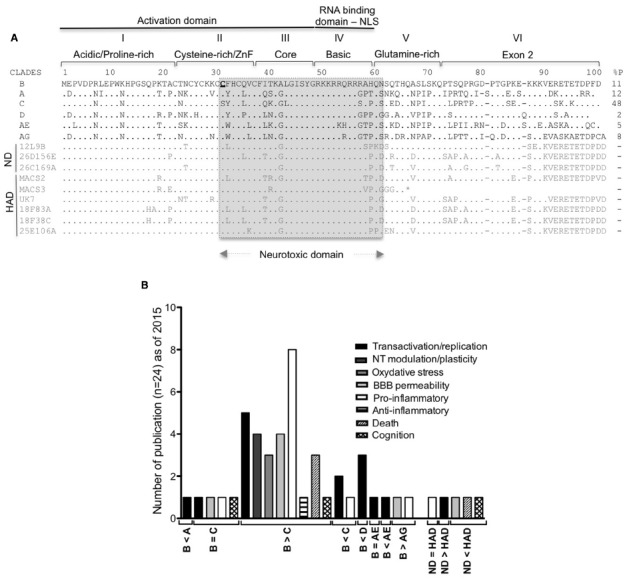
**Tat HIV clades and HAND. (A)** Conservation of the nucleotide sequences of Tat representative of the main HIV-1 clades (A–D and the circulating recombinant CRF_ AE/AG), brain derived isolates from non-demented HIV/AIDS individuals (ND sequences from Boven et al., 2007) and from individuals with HIV associated dementia [HAD sequences from Boven et al. (2007) and Thomas et al. (2007)]. Tat is encoded by two exons, divided into six functional regions. Region I (residues 1–21) is a proline-rich region, shown to protect Tat from degradation (Campbell and Loret, 2009; Caputo et al., 2009). Region II (residues 22–37) has seven conserved cysteines except for subtype C (which has ^31^C→^31^S) and the recombinant CRF_AE and CRF_AG (with more cysteines). Any mutation of these cysteines (except ^31^C) leads to loss of the transactivation activity (Kuppuswamy et al., 1989; Jeang et al., 1999). Region III (residues 38–48) has a conserved ^38^F(x)2KxLGISY motif. Mutation of ^41^K results in loss of transactivation (Kuppuswamy et al., 1989; Peloponese et al., 2000). ^38^F is conserved in Tat sequences and shown to be involved in binding to tubulin, resulting in apoptosis (Chen et al., 2002). Region I, II, and III constitute the minimal activation domain, which binds to cyclin T1. Region IV (residues 49–59) is rich in basic residues with the conserved sequence ^49^RKKRRQRRRPP. This domain is responsible for Tat’s interaction with TAR and is also a nuclear and nucleolar signal. Mutations in this domain results in loss of transactivation (Hauber et al., 1989) and delocalization of Tat from the nucleolus (Mousseau et al., 2012). The regions II as well as IV and Tat peptides covering the 31–61 amino acid region (in gray) were demonstrated to be neurotoxic (Mabrouk et al., 1991; Sabatier et al., 1991; Philippon et al., 1994; Weeks et al., 1995; Vives et al., 1997; Jones et al., 1998; Jia et al., 2001; Turchan et al., 2001; Self et al., 2004; Aksenov et al., 2006; Daily et al., 2006; Li et al., 2008; Mishra et al., 2008). Region V (residues 60–72) is the glutamine-rich region and was shown to be involved in apoptosis of lymphocytes T (Campbell et al., 2004). Regions I to V are encoded by exon I. Region VI is encoded by the second exon and contain a conserved RGD motif, found only in subtypes B and D. Predicted stop codon is indicated by an asterisk (*). % P: percentage of HIV-1 infected individuals in 2004–2007 with the indicated type of clade (Hemelaar et al., 2011). **(B)** Number of publications comparing the activity of HIV-1 clade B to other clades in the indicated neurotoxic activities.

The early HIV-1 gene product, Tat, is an 86–103 amino acid protein, divided in six functional regions (Kuppuswamy et al., 1989; Figure 1A). Tat binds through its basic domain to the 5′-terminal region of HIV mRNA’s stem-bulge-loop structure transactivation response element (TAR), and recruits the positive transcription elongation factor b (P-TEFb) to promote transcriptional elongation from the viral promoter (Cann et al., 1985). In addition to its key role in HIV-1 transcription amplification, Tat can be transported and secreted by HIV-1 infected cells, and can cross the BBB. Once in the brain, non-infected cells may uptake Tat passively or *via* interaction with membrane receptors (reviewed in Li et al., 2009). Immunostaining patterns suggest that Tat can be found in the cytoplasm of perivascular macrophages, microglia nodules and in glial cells, but also in the nuclei of some neurons and oligodendrocytes. These data suggest that Tat can be taken up by all CNS cells and potentially exert its effects distally from HIV-1 replication sites (Del Valle et al., 2000; Hudson et al., 2000; Liu et al., 2000). As noted above, the neurotoxic activity of Tat comes from both direct action on neurons and by altering the release of different soluble factors from surrounding non-neuronal cells resulting in neuronal or synaptodendritic injury. Brain histological changes similar to those observed in HAD patients have been observed in different mouse models expressing HIV-1 Tat (reviewed in Rappaport et al., 1999; Bruce-Keller et al., 2003; Chauhan et al., 2003; Kim et al., 2003). A positive correlation has also been shown between the levels of Tat mRNA transcripts and HIV- and simian human immunodeficiency virus (SIV)-induced encephalitis (Hudson et al., 2000).

The majority of the studies investigating neurotoxicity mediated by Tat demonstrated that the neurotoxic domain comprised part of the cysteine-rich/ZnF domain, the core and the basic domain of the protein (Figure 1A). Studies on HIV-1 clade B demonstrated the strong contribution of the highly conserved cysteine-rich/ZnF domain to neurotoxicity (Aksenov et al., 2009). In fact, studies carried out using other clades reported lesser neurotoxicity, which was attributed to differences in the sequence of the cysteine-rich/ZnF domain (Mishra et al., 2008). A summary of these findings is shown in Figure 1B. Interestingly, Tat from HIV-1 isolates found in patients with HAD exhibited higher neurotoxic potential than Tat isolated from non-HAD patients (Figure 1B). This suggests a relationship between characteristics of the brain environment and the selection of neurotoxic isolates, and validates the relevance of using Tat from virus present in HAD-positive individuals to study Tat activity, rather than laboratory adapted viruses. Regrettably, most of the studies on Tat to date do not address potential differences between the neurotoxic activity of Tat expressed upon transfection of eukaryotic cells and recombinant Tat produced in bacteria. Of the few studies that have offered such comparisons, either no differences were observed (Zauli et al., 2000; Koller et al., 2001), or higher toxicity was detected with transfected Tat when equivalent concentrations of both were examined (Li et al., 2008). These results could be explained by differences on the folding of Tat protein *in vitro*, and *in vivo*, or by differences of post-transcriptional modifications of Tat between eukaryotic and prokaryotic cells. There is also the possibility that the recombinant protein suffers some degradation when added to cell cultures, resulting in reduced activity.

### Tat and Neurotransmitters Modulation

The effects of Tat on neurotransmission have been largely studied on the glutamatergic (GLUTergic) and dopaminergic (DAergic) systems. Glutamate is an excitatory neurotransmitter essential for learning, memory, and synaptic plasticity (reviewed in Rahn et al., 2012), and has been shown to regulate drug reward (Campbell et al., 2004; Mishra et al., 2008). However, excessive glutamate causes “glutamate excitotoxicity” leading to neuron death (reviewed in Rahn et al., 2012). Tat protein impairs glutamate reuptake by astrocytes (Zhou et al., 2004) and increases microglial glutamate release (Gupta et al., 2010), elevating extracellular glutamate levels. The down-regulation of the excitatory amino acid transporter-2 (EAAT-2) involved in the reuptake of glutamate and the stimulation of the glutamate release from microglial cells, was also reported in the presence of Tat (Rumbaugh et al., 2007). More directly, Tat further promotes glutamate excitotoxicity by directly binding to or phosphorylating the glutamate *N*-methyl-D-aspartate (NMDA) receptor (Haughey et al., 2001; Prendergast et al., 2002; Song et al., 2003). The Cys^30–31^ motif in the clade B Tat protein interacts with the extracellular domain of the NR1 subunit of the NMDA receptor (Li et al., 2008). The mutation of Cys^31^ into Ser^31^ in HIV-1 Tat expressed by HIV-1 clade C and/or polymorphisms of the NMDA receptor subunits could explain the lesser neurotoxic activity of clade C Tat (Mishra et al., 2008; Rao et al., 2008; King et al., 2010; Eugenin et al., 2011). Interestingly, a recent study showed that Tat potentiates NMDA-evoked increases in intracellular Ca^2+^ concentration ([Ca^2+^]i) *via* the low-density lipoprotein receptor-related protein (LRP) and activation of Src tyrosine kinase, and mediated by a GTPase RhoA and Rho-associated protein kinase (ROCK; Krogh et al., 2015). However, potentiation is rapidly followed by a gradual adaptation and decrease of [Ca^2+^]i, resulting in neuronal protection from excessive calcium influx (Krogh et al., 2014, 2015). This suggests that even if Tat activates the NMDA receptor at picomolar concentrations (Li et al., 2008), Tat-mediated neurotoxicity through NMDA may require a certain activation threshold. Consistent with this, Tat inhibits glutamate-induced variation in intracellular Ca^2+^ in astrocytes (Koller et al., 2001), resulting in protection from death. Several studies using astrocyte cells to study the effects of Tat in HAND and/or the response to drugs of abuse may take advantage of this protection from death (Chauhan et al., 2003; Kim et al., 2003; Thomas et al., 2007; Mediouni et al., 2015).

The neurotransmitter dopamine (DA) plays important roles in motor control, motivation, reward, arousal, cognition, executive functions, and maternal behaviors (reviewed in Nutt et al., 2015). The impairment of DA neuronal functions correlates with early stage of HIV infection (Berger et al., 1994). Tat perturbs the normal signaling of DA transmission by inhibiting the activity of the DA transporter (DAT) through a concentration-dependent allosteric mechanism (Chan and Kantor, 2009; Zhu et al., 2009) without affecting DA release (reviewed in Turowski and Kenny, 2015). This prevents astrocytes from buffering DA and from triggering the release signal for neuroprotective molecules mediated through DAT (Silvers et al., 2007; Zhu et al., 2009, 2011; Perry et al., 2010; Miyazaki et al., 2011; Midde et al., 2012, 2013). Tat also inhibits the vesicular monoamine transporter (VMAT2) and the expression of tyrosine hydroxylase (TH, the rate limiting enzyme in DA biosynthesis), thus amplifying DA neurotoxicity (Zauli et al., 2000; reviewed in Everall et al., 2005; Midde et al., 2012, 2013; Theodore et al., 2012). These findings, associated with disturbances in the DAergic system are observed both in humans and in animals models (reviewed in Koutsilieri et al., 2002a,b; Jenuwein et al., 2004) and may have implications in the development of symptoms commonly observed in both Parkinson’s disease and in HAND (reviewed in Koutsilieri et al., 2002a,b).

### Tat and Oxidative Stress

Low- and short-term reactive oxygen species (ROS) such as hydrogen peroxide (H_2_O_2_) and nitric oxide (NO) are important to cellular physiology as they participate in cell signaling in several biological processes (reviewed in D’Autreaux and Toledano, 2007; reviewed in Erusalimsky and Moncada, 2007). In the brain, ROS and NO interact in many ways to induce activation of brain cell glial subpopulations (Saha and Pahan, 2006a,b) and promote neuronal activity (Atkins and Sweatt, 1999). However, sustained high levels of ROS induce lipid peroxidation, protein oxidation and impairment of DNA synthesis and fidelity, events collectively called “oxidative stress” (reviewed in Pocernich et al., 2005; reviewed in Schieber and Chandel, 2014). Markers of oxidative stress are detected in brain and cerebrospinal fluid (CSF) of HAD patients (reviewed in Pocernich et al., 2005). Oxidative stress and neurodegeneration are secondary to glial activation and occur when ROS and NO approach inflammatory levels.

Tat has been shown to alter the production of ROS, in particular of reactive nitrogen species, leading to a loss of neuronal activity. Several studies *in vivo* and *in vitro* have shown an increase in the expression of intracellular NO synthase (NOS2) in brain cell subsets, resulting in an increase of NO following Tat exposure (Philippon et al., 1994; Bukrinsky et al., 1995; Jones et al., 1998; Liu et al., 2002; Kim et al., 2015). Excess NO may induce glutamate release from astrocytes (Bal-Price et al., 2002), thereby further hyperactivating NMDA receptors on neurons that, in turn, triggers further ROS-associated neuronal death (Capone et al., 2013). Moreover, in astrocytes exposed to Tat, a NOS2-dependent mechanism up-regulates adhesion molecules that induce recruitment of monocytes and viral replication (Song et al., 2011), consistent with the observation that elevated NO levels are associated with increased HIV-1 replication (reviewed in Torre et al., 2002). In parallel, Tat can induce the expression of factors that regulate its own activity over the HIV LTR, such as Nrf2 (nuclear erythroid 2 related factor 2). Nrf2 is a critical regulator of genes involved in the antioxidant response. Although the induction of Nrf2 by Tat is not enough to protect cells from oxidative stress in MAGI cell systems (Zhang et al., 2009), the ROS-induced expression in astrocytes has been reported to show a strong neuroprotective effect (Bell et al., 2011).

Other markers of oxidative stress such as protein carbonyl formation and lipid peroxidation *in vitro* and *in vivo*, as well as perturbation of the cellular antioxidant response have also been linked to Tat expression (Flores et al., 1993; Milani et al., 1996; Borgatti et al., 1998; Haughey et al., 1999, 2004; Choi et al., 2000; Aksenov et al., 2001; Richard et al., 2001; Pocernich et al., 2004; Agrawal et al., 2012; Kim et al., 2015).

### Tat and Blood–Brain Barrier Disruption

The BBB is composed of brain endothelial cells connected by tight junctions and is supported by astrocytes. Tat and specific Tat peptides can passively cross the BBB in both directions (Banks et al., 2005). Tat can also use different mechanisms to decrease the expression of tight junction proteins and basement membrane extracellular matrix proteins by promoting the expression of inflammatory molecules, oxidative stress and the expression of the matrix metalloproteinase 9 (MMP-9, a zinc dependent endopeptidase; reviewed in Yong et al., 1998; Zidovetzki et al., 1998; Weiss et al., 1999; Pu et al., 2007; Ju et al., 2009; Banerjee et al., 2010; Song et al., 2011; Xu et al., 2012; Huang et al., 2014). Consistent with these effects, agonists of the peroxisome proliferator activated receptors (PPAR), a group of a nuclear receptor proteins mediating expression of anti-inflammatory and anti-oxidative genes, reduce Tat mediated BBB disruption in mice (Huang et al., 2014).

The level of BBB damage may vary depending on the Tat sequence variant found in different clades of the virus. Tat from the HIV-1 B subtype and Tat from isolates recovered from patients diagnosed with advanced HAD proved the most potent in disrupting the BBB (Figure 1B).

Of the molecules present on cells of the BBB that change upon exposure of Tat protein, P-glycoprotein (P-gp) is critical, since it is a pump involved in the transport of small molecule compounds out of the brain. Interestingly, the efflux function, expression and promoter activity of P-gp are up-regulated by Tat (Zhong et al., 2010). This may help explain why HIV-infected individuals maintained on suppressive therapy present low levels of ART in the brain (Gimenez et al., 2004; reviewed in Miller et al., 2008), with up-regulated P-gp removing ART from the CNS. A better understanding of the mechanism of action of Tat on P-gp may offer solutions to improve the efficiency of ART in the brain reservoir of HIV, not only for the enhancement of ART crossing the BBB, but also for improving the anti-viral performance of immune cells that express this protein (reviewed in Owen and Khoo, 2004).

### Tat, Glial Cells, and Neuroinflammation

Markers of gliosis have been found *in vitro* after treatment of cells with either Tat or its neurotoxic domain. The same is true *in vivo* in the brain and CSF of HAD-affected individuals (Gallo et al., 1989; Tyor et al., 1992; Glass et al., 1993; Wesselingh et al., 1993; Philippon et al., 1994; Jones et al., 1998; Shi et al., 1998; Bruce-Keller et al., 2003; Kim et al., 2003; Turchan-Cholewo et al., 2009a,b; Puccini et al., 2015). Indeed, Tat induces the expression of important pro-inflammatory cytokines such as tumor necrosis factor α (TNF-α), interleukin-1β (IL-1β) and interleukin-6 (IL-6) (Ambrosino et al., 1997; Chen et al., 1997; Zidovetzki et al., 1998; Nath et al., 1999; Buscemi et al., 2007; reviewed in Li et al., 2010; Williams et al., 2010; Yang et al., 2010; Zhao et al., 2014; Mediouni et al., 2015), which make strong contributions to the development of an inflammatory neuropathology. For instance, TNF-α is associated with increased HIV-1 replication, NMDA receptor neurotoxicity and BBB disruption (Wright et al., 1988; Merrill et al., 1989; Stone, 1993; Fine et al., 1996; Heyes et al., 1996; Nuovo and Alfieri, 1996; Bezzi et al., 2001; Smith et al., 2001; reviewed in Brabers and Nottet, 2006; Buscemi et al., 2007; Wang et al., 2008; Zhong et al., 2008; Ju et al., 2009; Kiebala et al., 2010; Kiebala and Maggirwar, 2011). Likewise, IL-1β up-regulation, which leads to the transcription of several pro-inflammatory cytokines and chemokines, has been correlated with the severe neurologic symptoms of HAD patients (Gallo et al., 1989; reviewed in Allan et al., 2005; reviewed in Moynagh, 2005; reviewed in Brabers and Nottet, 2006). In the pathway that leads to Tat up-regulation of these two pro-inflammatory cytokines, targeting of the protein-tyrosine phosphatase (PTP), or CD45, was shown to attenuate microglia activation, suggesting a promising drug target (Jin et al., 2012). Additionally, elevation of IL-6 levels was reportedly associated with increased BBB permeability (Zidovetzki et al., 1998) and HIV replication (reviewed in Li et al., 2010). Tat-induced expression of inflammatory cytokines was also shown to involve other potential targets, such as the Ankyrin-rich membrane spanning protein (ARMS/Kidins220) in microglia cells (Singh et al., 2015) and the regulator of microglial phagocytosis (Leucine-rich repeat kinase 2; Marker et al., 2012).

In addition to the effect on cytokines, Tat modulates chemokine pathways in microglia, inducing neuroinflammation and changes in BBB permeability. Tat may bind to chemokine receptors through its cysteine/ZnF domain (a chemokine like sequence), thereby inhibiting the expression of CX3CR1 by suppressing a NF-kB-YY1 pathway, or inducing the expression of the potent chemoattractant chemokine MCP-1 (Fuentes et al., 1995; Albini et al., 1998; Weiss et al., 1999; Eugenin et al., 2003; Sheng et al., 2003; Bethel-Brown et al., 2011; Duan et al., 2014; Tewari et al., 2015). However, the induction MCP-1 by Tat may result in a complicated mode of action, since it has also been shown that MCP-1 protects both neurons and astrocytes from NMDA or Tat-induced apoptosis (Eugenin et al., 2003).

### Tat and Apoptotic Pathways

As Tat influences processes of excitotoxicity, neuroinflammation and oxidative stress, it also modulates intrinsic cellular apoptotic pathways to variably promote both cellular death or protection. Apoptosis induced by Tat may be triggered by different mechanisms such as the disturbance of microtubule functions, changes of mitochondrial activity, reduction of the activity of the tumor suppressor p53 (involved in neuronal differentiation and survival), induction of reticulum stress, or triggering of the complex LRP/postsynaptic density protein-95/NMDA receptor/neuronal nitric oxide synthase (nNOS) at the neuronal membrane (reviewed in Roshal et al., 2001; Chen et al., 2002; Campbell et al., 2004; Pourroy et al., 2004; de Mareuil et al., 2005; Eugenin et al., 2007; Norman et al., 2007; Darbinian et al., 2008; Lecoeur et al., 2012; Liu et al., 2014; Ma et al., 2014b). On the other hand, infected cells from HIV infected patients Tat plays a critical role inducing pathways that inhibit autophagy, phagocytosis, or the expression of MHC molecules, as well as up-regulation of p73 (Chehimi et al., 1996; reviewed in Huigen et al., 2004; Amini et al., 2005; Saunders et al., 2005; reviewed in Li et al., 2010; Van Grol et al., 2010; Hui et al., 2012; Debaisieux et al., 2015). Thus, it is conceivable that Tat may cause the death of cells such as neurons and supportive glial subsets, while perversely preserving host infected cells, thereby contributing to the status of the brain as a reservoir at the cost of neurodegeneration.

## Methamphetamine Neurotoxicity

**FIGURE 2:**
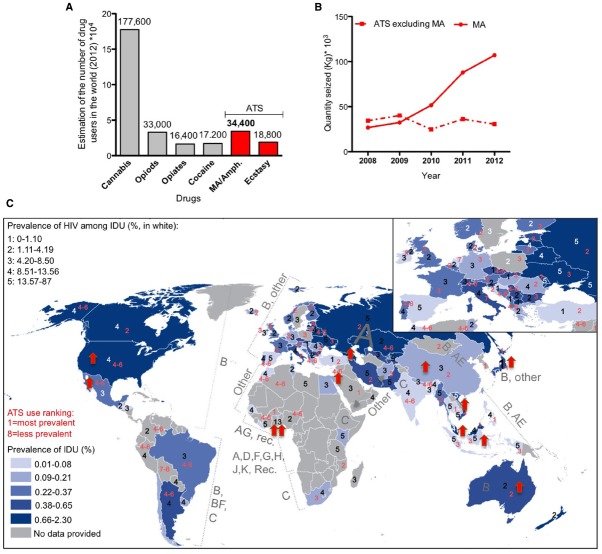
**Worldwide distribution of methamphetamine (MA) use and HIV infection. (A)** Worldwide estimation of drug use in 2012 (data from the world drug report 2014). ATS: amphetamine-type stimulants. **(B)** Total amount of seized drugs reported worldwide 2008–2012 (data from UNODC global synthetic drugs assessment 2014). **(C)** Superimposition of maps of the prevalence of intravenous drug use (IDU, map from the world drug report 2014) in 2012 with the prevalence of HIV infection among IDU in 2012 (numbers in white, data from the world drug report 2014), completed with the world distribution of HIV clades (data from Chan and Kantor, 2009) and the ranking of ATS use in 2012 (numbers in red, data from the world drug report 2014). Red arrow corresponds to an increase of more than 10% in MA/amphetamine drug use in 2011–2012 in the indicated country (data from the drug world report 2014).

Methamphetamine belongs to the amphetamine-type stimulant family. Used clinically at low doses to improve behavioral and cognitive functions after brain injury (Rau et al., 2012), MA is a strong psychostimulant that is widely abused as a “street drug.” Easily synthesized, relatively long lasting, and generally inexpensive in comparison to cocaine and heroin, MA is the second most commonly abused drug in the world (Figure 2A). Although MA seizure has increased dramatically in the last few years (Figure 2B), these factors have contributed to the increase of drug-associated health problems and social burden.

Methamphetamine induces acute euphoria, the perception of limitless energy and high libido. Physiologically, it induces potentially lethal hyperthermia, due to both CNS and peripheral effects (Sanchez-Alavez et al., 2014). Chronic MA abuse leads to neuropsychiatric complications including memory impairment, attention deficit and psychomotor decay associated with neurotoxicity and neurological dysfunction, as well as significant dysregulation of neurotransmitters levels. Importantly, MA use correlates with enhanced risk-taking behaviors that may place drug abusers at a higher risk of exposure to infections such as Hepatitis C and HIV, each further contributing to CNS dysfunction. The impressive epidemiological overlap between intravenous drug use (IDU) and the population that is HIV-positive worldwide confirms this trend (Figure 2C).

### Methamphetamine and Neurotransmitter Modulation

Although MA modulates a broad assortment of neuro-transmitters, MA-induced toxicity is largely attributed to hyperactivation of the DAergic system. MA increases extracellular DA levels, resulting in long-term degeneration of DAergic neurons, a mechanism also responsible for the motor impairments associated with Parkinson’s disease (reviewed in Riddle et al., 2006). MA exposure also promotes cognitive deficits related to DAergic neurodegeneration (reviewed in Riddle et al., 2006). Considerable neurochemical evidence of this neurotoxicity includes the demonstrated decrease of DAT and the activity of TH, a decrease of degradation of DA by monoamine oxidase (MAO), a reduction of activity and protein levels of VMAT2, and an increase of the reverse transport of DA *via* the DAT into the extracellular space (Gibb and Kogan, 1979; Zaczek et al., 1991; Giros et al., 1996; reviewed in Riddle et al., 2006; Eyerman and Yamamoto, 2007; Xie and Miller, 2009; reviewed in Silverstein et al., 2011; reviewed in Coller and Hutchinson, 2012). MA acts through the DA receptors (D1 and D2) and the TAAR1 receptor, but recent studies have also posited a role for other DA receptors specially the D3 involved in addiction, hyperthermia and modulation of the immune system (Ricci and Amenta, 1994; Ricci et al., 1997, 1998; Amenta et al., 1999; Ricci et al., 1999; Granado et al., 2011a, Ares-Santos et al., 2012; reviewed in Yu et al., 2015).

Following the increase of extracellular DA, MA causes a rise in striatal glutamate release (Nash and Yamamoto, 1992; Stephans and Yamamoto, 1994; Mark et al., 2004). Glutamate receptors, the EAAT-2 and VGLUT (the vesicular glutamate transporters, which are in charge of transporting glutamate into synaptic vesicles), are also involved in mediating different behaviors attributed to MA, such as reward, hyperlocomotor activity and, to some extent, hyperthermia (Golembiowska et al., 2003; Achat-Mendes et al., 2012a,b; reviewed in Cisneros and Ghorpade, 2012; Crawford et al., 2013; Herrold et al., 2013; He et al., 2014; Hiyoshi et al., 2014; Suryavanshi et al., 2014).

Independent from the DAergic system, MA also directly modulates the presynaptic release of glutamate in rat cultured hippocampal primary neurons (Zhang et al., 2014). Furthermore, serotonergic, γ-amino-butyric acid-ergic (GABAergic) and cholinergic systems are also altered by MA, highlighting the widespread damage caused by this drug (Halpin et al., 2014; reviewed in Northrop and Yamamoto, 2015; reviewed in Yu et al., 2015).

### Methamphetamine and Oxidative Stress

Several *in vivo* and *in vitro* studies showed the increase of cytoplasmic and mitochondrial oxidative stress markers following acute or chronic exposition to MA (reviewed in Silverstein et al., 2011; Sanchez-Alavez et al., 2013; Halpin et al., 2014; Nam et al., 2014; Sanchez-Alavez et al., 2014; Solhi et al., 2014; Walker et al., 2014; reviewed in Yu et al., 2015). Importantly, several neurotoxicity hallmarks induced by MA are absent in animals that express the human superoxide dismutase 1 (SOD 1) gene, or that can otherwise quickly detoxify free radicals induced by MA (Cadet et al., 1994; Hirata et al., 1995, 1996, 1998; Deng and Cadet, 2000; Deng et al., 2001). In addition, antioxidant treatments have shown neuroprotective effects against MA-induced brain damage, suggesting a significant role for oxidative stress in MA toxicity (reviewed in Chen et al., 2010; Lecoeur et al., 2012; Barayuga et al., 2013; Koriem et al., 2013; reviewed in Liddie et al., 2013; Shin et al., 2014). Antioxidants are also able to prevent and reverse MA-induced hyperthermia, and MA-associated mitochondrial changes in animal models, suggesting that the thermal dysregulation in drug abuse, which is also reported as a trigger of neurotoxicity, involves the capacity of MA to induce ROS (Sanchez-Alavez et al., 2013, 2014).

The effects of MA are associated with cellular energy consumption and increased metabolism, potentially leading to mitochondrial dysfunction. Mitochondria functions are affected by the inhibition of ATP and the complex II of the electron transport chain (Burrows et al., 2000; Brown et al., 2005), mediated both by glutamate and NO (Brown et al., 2005). Moreover, ROS and quinone, a product of DA oxidation, are both known to inhibit mitochondrial enzymes associated with energy production (O’Shea et al., 2014). Therefore, the oxidative stress that results from MA abuse may have dramatic consequences for mitochondria metabolic efficiency.

Interestingly, unlike Tat, MA-induced astrogliosis was associated with the decrease of Nrf2 (Granado et al., 2011b), suggesting that astrocytes may have a low antioxidant capacity when exposed to MA.

### Methamphetamine and Blood–Brain Barrier Disruption

Several preclinical and clinical studies reported BBB dysfunction by MA through different mechanisms (reviewed in Kousik et al., 2012; Matsumoto et al., 2014; reviewed in Northrop and Yamamoto, 2015; reviewed in Turowski and Kenny, 2015). MA-induced oxidative stress, hyperthermia, glutamate release and induction of inflammation combine to decrease tight junctions and transendothelial electric resistance *via* oxidative stress, down-regulate MMP expression mediated by hyperthermia, activate glutamate receptors, and increase the release of inflammatory cytokines as well as the expression of heat shock proteins (Ramirez et al., 2009; reviewed in Silverstein et al., 2011; reviewed in Kousik et al., 2012; Shah et al., 2012; Martins et al., 2013; Urrutia et al., 2013; reviewed in Northrop and Yamamoto, 2015). In addition, MA exposure has been correlated with a lack of the glucose-induced energy required for tight junction assembly and in the attenuation of brain capillary endothelial cell growth (Abdul Muneer et al., 2011; Fisher et al., 2015). Taken together, these mechanisms account for collective insults to the BBB that precedes neurological symptoms associated with chronic MA abuse.

### Methamphetamine, Glial Cells, and Neuroinflammation

Modifications to the inflammatory status of CNS glial cells caused by MA exposure have strong implications that vary from biochemical alterations to behavioral changes. For instance, the release of cytokines by activated glial cells following MA exposure is correlated with behavioral effects such as high motor activity, MA self-administration and relapse, as well as hyperthermia (Boven et al., 2007; reviewed in Beardsley and Hauser, 2014; reviewed in Loftis and Janowsky, 2014; reviewed in Salamanca et al., 2014; reviewed in Yu et al., 2015). MA-induced DA and glutamate release can induce the activation of pattern recognition receptors (PRRs) in microglia that were shown to activate glial cells (Kuhn et al., 2006; reviewed in Beardsley and Hauser, 2014; Halpin et al., 2014; reviewed in Loftis and Janowsky, 2014).

Sigma receptors on glia and neurons have been recently implicated in MA-induced neuroinflammation. The sigma receptor is a chaperone protein at the endoplasmic reticulum that controls calcium signaling through the inositol triphosphate receptor. Various sigma receptor antagonists with different characteristics and potency have been characterized, and proven effective against neuroinflammatory activity (Kaushal et al., 2013, 2014; Zhang et al., 2015).

It has been suggested that voltage-gated potassium channels (Kv) are involved in neuronal damage as well as microglial function. Indeed, Kv1.3 is a major K^+^ channel expressed in microglia that has been previously involved in MA-induced microglia damage. MA significantly increases outward K^+^ currents and Kv1.3 antagonists increase microglial viability, while also decreasing the expression of neurotoxins and pro-inflammatory cytokines from these cells (Wang et al., 2014).

Interestingly, the pattern of microgliosis in individuals with a history of MA addiction has been shown to persist for at least 2 years into abstinence (Sekine et al., 2008). Likewise, astrocytes remain reactive up to 30 days following MA exposure. It is possible this is initially beneficial, turning maladaptive over time. In the short term, these effects may serve as a protection mechanism against MA-induced neurotoxicity (Hebert and O’Callaghan, 2000; Friend and Keefe, 2013; Robson et al., 2014). The release of neurotrophic factors, anti-inflammatory cytokines, and radical scavengers by glial cells to protect neurons from MA, have also been reported (reviewed in Beardsley and Hauser, 2014).

### Methamphetamine and Apoptotic Pathways

The interaction between the excitotoxicity, oxidative stress, neuroinflammation and BBB disruption results in significant neuronal death in DA-rich regions (reviewed in Coller and Hutchinson, 2012; reviewed in Loftis and Janowsky, 2014; reviewed in Salamanca et al., 2014; reviewed in Yu et al., 2015). The mechanisms of apoptosis can be either caspase-dependent or -independent (Jayanthi et al., 2004). Even though MA-mediated neuronal apoptosis is very well documented, the apoptosis of other cells in the brain is less clearly established (Deng et al., 2001; Jayanthi et al., 2004; Halpin et al., 2014). MA induces the death of glial and oligodendrocytes through caspase-dependent pathways (Genc et al., 2003; Jumnongprakhon et al., 2014). Melatonin has been shown to rescue glial cells from death (Jumnongprakhon et al., 2014), an effect that might be anticipated because melatonin serves several immune-related functions such as the modulation of immune cell numbers, inhibition of pro-inflammatory cytokines, increased neurogenesis and a reduction of adhesion molecules (reviewed in Maldonado et al., 2009). As such, melatonin was suggested as a potential candidate to treat mental disorders and to possess potential in MA recovery therapies (reviewed in Maldonado et al., 2009).

On the other hand, MA was also associated with the autophagy process to protect neurons and endothelial cells (Castino et al., 2008; Fornai et al., 2008; Lin et al., 2012; Ma et al., 2014a). Interestingly, caffeine increases MA induced toxicity in neuroblastoma cell lines by inhibiting this autophagy process (Sinchai et al., 2011; Pitaksalee et al., 2015).

## Tat and Methamphetamine Neurotoxicity

It is not surprising that a positive correlation exists between worldwide MA drug statistics (World drug report 2014) and the prevalence of HIV among intravenous drug users (Figure 2C). Interestingly, there is an increased tendency to MA/amphetamine consumption in countries that are predominantly affected with HIV-1 B subtype (Figure 2C), reported to be more neurotoxic (Figure 1B). Below we review some of studies that show Tat neurotoxicity enhanced by MA co-incubation.

### Tat/Methamphetamine and HIV Replication

One important activity of MA in HIV disease progression is the ability to enhance viral replication. MA was shown to activate the promoter of HIV-1 in microglia cells, which Tat synergistically amplifies (Wires et al., 2012).

Methamphetamine dysregulation of the DA system augments viral production in different immune cells by promoting HIV entry and HIV transcription (Rohr et al., 1999a,b; Reynolds et al., 2007; Liang et al., 2008; Toussi et al., 2009; Marcondes et al., 2010; Nair and Saiyed, 2011; reviewed in Purohit et al., 2011). A review by Purohit et al. (2011) nicely illustrates the effects of DA in HIV transcription caused by oxidative stress and NF-κB activation in the presence of drugs of abuse in the brain (reviewed in Purohit et al., 2011). Inhibition of phagocytosis, disruption of pH and the reduced expression of Toll-like receptor 9 have each been proposed as mechanisms to explain the MA promotion of spreading HIV infection (Talloczy et al., 2008; Cen et al., 2013). In turn, the MA-induced elevation in DA levels seems to induce the migration of activated monocytes into the brain in the context of HIV (reviewed in Gaskill et al., 2013; Coley et al., 2015). This finding may be also correlated to the ability of MA in non-human primate to up-regulate C-C chemokine receptor type 5 (CCR5), the co-receptor for viral entry, in microglia cells, and in brain-derived macrophages which otherwise do not exhibit a classical inflammatory phenotype based on surface markers (Marcondes et al., 2010).

An interesting pathway with ties to the neuroinflammatory process triggered by MA that modulates infection is the Wnt/β-catenin signaling cascade, which regulates the survival process of neurons and astrocytes. In fact, astrocytes are the primary source of Wnt ligands, triggering the Wnt signaling cascade that mediates anti-inflammatory responses, neuroprotection and neurogenesis (Sharma et al., 2011). It has been reported that the Wnt/β-catenin/transcription factor 4 signaling pathway inhibits HIV replication in astrocytes. Co-incubation of primary human astrocytes with Tat and MA leads to an increased down-regulation of the Wnt signaling by more than 50% compared to the activity of MA or Tat alone. Given the additive effect of MA and Tat, it was suggested that the toxins may be engaging distinct pathways (Sharma et al., 2011).

### Tat/Methamphetamine and Neurotransmitters Modulation

The effects of Tat and MA on neurotransmission have been described primarily in the context of the DA and GLUTergic systems. Cooperative effects of Tat and MA have been shown to enhance the DA mediated amplification of viral replication in correlation with the enhanced motor-stimulant response to a recurrent exposure to MA, a demonstration of behavioral sensitization (Liu et al., 2009). Moreover, Tat and MA showed *in vivo* a synergetic effect on the death of striatal DA terminals (Maragos et al., 2002; Cass et al., 2003; Theodore et al., 2006b,d; reviewed in Theodore et al., 2007).

Pre-pulse inhibition (PPI) is a neural mechanism in which a weaker pre-stimulus (pre-pulse) reduces the reaction to a following strong startling stimulus (pulse), and is used to measure adaptive inhibition mechanisms. This is thought to be a measure of sensorimotor gating and deficits have been reported in schizophrenia (Bertrand et al., 2014).

Patients with HAND demonstrate deficits in PPI responses not observed in HIV-positive participants without neurocognitive impairment (Greenwood et al., 2007). Similarly, intra-hippocampal infusion of Tat protein disrupts acoustic startle response and PPI in rats (Nath and Steiner, 2014). Among adult HIV-1 transgenic rats (that express seven of the nine clade B HIV-1 genes), Tat has a synergistic role in the increase of the MA-mediated disruption of PPI, an effect attributed to its ability to decrease TH while increasing MAO (Moran et al., 2012). Together, these findings suggest disruptions of DAergic signaling with behavioral and cognitive consequences.

In the GLUTergic system, the combination of MA and Tat was shown to be additive in enhancing the disruption of EAAT-2 in astrocytic cells. This leads to elevated glutamate levels in the synaptic clefts and a synergistic increase in midbrain neuronal death *in vitro*, in a process that requires both D1 and NMDA-receptors (Aksenov et al., 2012; reviewed in Cisneros and Ghorpade, 2012).

### Tat/Methamphetamine and Oxidative Stress

Co-incubation of Tat and MA in cell culture *in vitro* has an additive effect promoting oxidative stress (Langford et al., 2004). Likewise, the combination *in vivo* results in a synergic/additive activation of redox system-regulated transcription factors, the increased expression of the intracellular adhesion molecule-1 (ICAM-1) gene, and of cytokines (TNF-α and IL-1β) in some brain regions (Flora et al., 2003). Tat and MA also have an additive effect in decreasing antioxidant enzyme levels (Banerjee et al., 2010). Other studies have shown that the combination of the HIV-1 envelope glycoprotein 120 (gp120) with Tat/MA also has the capacity of synergistically reducing the expression of tight junction proteins through a mechanism that seems to be dependent on oxidative stress, resulting in an impairment of the BBB (Banerjee et al., 2010). This report also demonstrates the possible synergy between the viral proteins in the induction of oxidative stress (Banerjee et al., 2010). On the other hand, a recent study has shown no significant concerted effects between HIV and substance of abuse in the increase of oxidative stress markers detected in the CSF of HIV-positive subjects, with or without substance use disorders (Panee et al., 2015). The differences between these studies may be due to different levels of Tat and/or MA used in the *in vitro* experiments and models, particularly in comparison to how much Tat is present in patients and their rate of MA usage. Tat in the serum of HIV-infected patients was detected at about 40 ng/ml (Xiao et al., 2000), and the concentration of MA in postmortem brains of chronic MA abusers is reportedly around 0.8–1 mM (Talloczy et al., 2008). However, relatively little work has been done on this topic, and the magnitude of the progression of HIV infection, the frequency of drug use, the composition of ART and the sensitivity of the assays may all influence the outcome of these studies.

### Tat/Methamphetamine and Blood–Brain Barrier Disruption

Tat and MA synergistically and additively alter the BBB through the increase of cell adhesion molecules (ICAM-1), the decrease of the tight junction proteins and induction of MMP (Flora et al., 2003; Conant et al., 2004; Banerjee et al., 2010). Moreover, Kass et al. (2010) reported that MA-induced hyperthermia was enhanced in HIV transgenic rats (Kass et al., 2010), consistent with hyperthermia-induced BBB permeability (reviewed in Northrop and Yamamoto, 2015). It has been proposed that low non-toxic doses of Tat and MA could potentially be helpful in temporarily reducing the BBB permeability to enhance the access of antiretrovirals to the CNS (reviewed in Turowski and Kenny, 2015).

### Tat/Methamphetamine, Glial Cells, and Neuroinflammation

As previously mentioned, Flora et al. (2003) showed that co-administration of Tat and MA resulted in the higher expression of the pro-inflammatory cytokines TNF-α and IL-1β, as well as the adhesion molecule ICAM-1 in several mouse brain regions (Flora et al., 2003). Theodore et al. (2006c) confirmed the involvement of TNF-α in depletion of DA levels, using a double KO for TNF-αR1 and TNF-αR2. The induction of TNF-α was also associated with a greater loss of hippocampal neurons (Theodore et al., 2006c,d).

A cytokine array performed on rat striatal tissue treated with a combination of MA and Tat revealed synergistically higher levels of MCP-1, timp metallo-peptidase inhibitor 1 (TIMP-1), and IL-1α. This activity was absent in MCP-1 KO mice, highlighting the importance of MCP-1 in Tat/MA neurotoxicity (Theodore et al., 2006a). These findings strongly suggest a crucial role for cytokines in mediating the interactions between MA and Tat in HIV-associated neurotoxicity.

Sharma et al. (2011) previously described the additive effect of Tat and MA in down-regulating the Wnt/β-catenin survival pathway (Sharma et al., 2011). In that study, the authors found that unlike Tat, MA also down-regulated NF-κB reporter activity, and as expected, decreased MCP-1 and IL-6 mRNAs. When Tat and MA were used in combination, the reduction of NF-κB reporter activity and MCP-1 mRNA persisted, but IL-6 expression was normalized. This seeming discrepancy could be explained by the fact that Tat can transactivate the IL-6 promoter by binding to IL-6 leader RNA (a stem loop structure located at the 5′-terminal region of the IL-6 mRNA) and by interacting and increasing the CAAT enhancer-binding protein (C/EBP) DNA binding activity (NF-IL-6) at the IL-6 promoter (reviewed in Li et al., 2010). Together, this suggests that the effect of both toxins together is not always positively correlated, as each toxin could trigger different pathways and produce unexpected phenotypes.

### Tat/Methamphetamine and Apoptotic Pathways

The cooperation between Tat and MA in triggering BBB permeability, oxidative stress and increased viral replication may explain the severity of HAD symptoms in HIV-infected MA abusers, as compared to non-drug abusing patients (Nath et al., 2001; Sporer et al., 2005; reviewed in Ferris et al., 2008; reviewed in Purohit et al., 2011; reviewed in Silverstein et al., 2011; reviewed in Strazza et al., 2011; reviewed in Purohit et al., 2013; reviewed in Hauser and Knapp, 2014). This is confirmed in animal studies using the fully replicating virus in combination with MA, showing greater alteration of cognitive and locomotor behavior when compared with either treatment alone (Liu et al., 2009; Kass et al., 2010; reviewed in Silverstein et al., 2011; Kesby et al., 2015), an effect mediated by inflammation and oxidative stress processes (Maragos et al., 2002; Theodore et al., 2006a,b,c,d; Banerjee et al., 2010). The cognitive and locomotor decay may also be explained by the synergic activity of Tat and MA in triggering autophagy and apoptosis mechanisms. For instance, in presence of Tat and MA an increase of autophagosomes and/or multilamellar bodies was observed upon death of a neuroblastoma cell line (Cai and Cadet, 2008; Qi et al., 2011).

Melatonin has been shown to rescue glial cells from death and it was proposed to have potential in MA recovery therapies (reviewed in Maldonado et al., 2009; Jumnongprakhon et al., 2014). Tat was shown to up-regulate melatonin (Kaushal et al., 2014), with potentially resulting effects that remain to be assessed.

### Caveats

**FIGURE 3:**
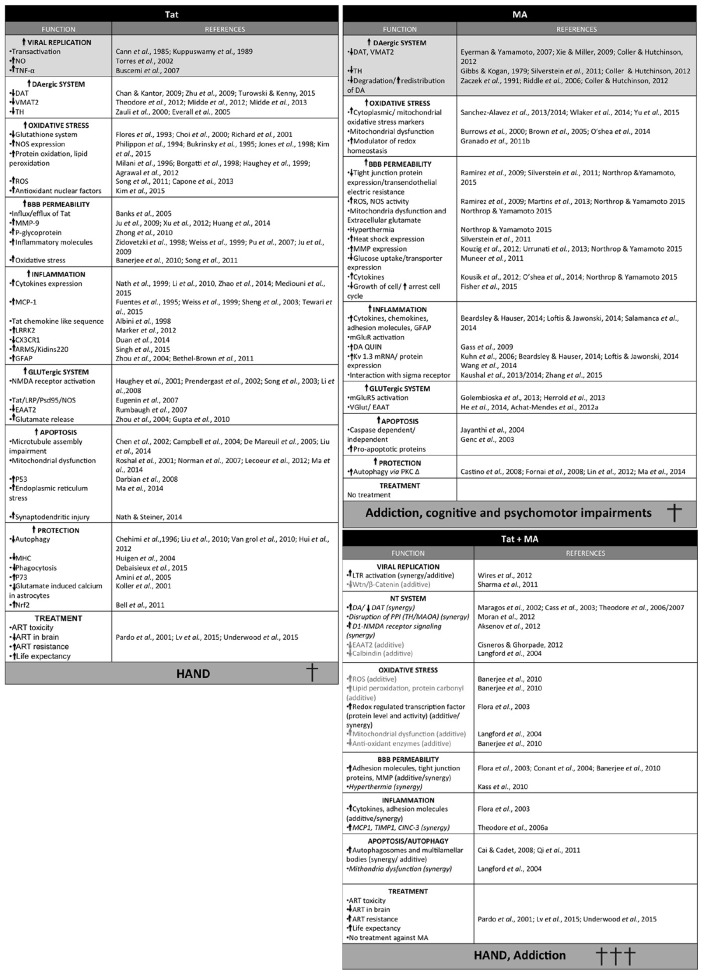
**Summary of the different effects of Tat, methamphetamine (MA) or Tat and MA in combination.** Synergic activity is shown in italic and additive effects in light gray. Light gray squares correspond to the main activity of each toxin.

Throughout this review, we have discussed different mechanisms by which Tat and MA may mediate neurocognitive and locomotor disorders (summarized in Figure 3). Nevertheless, these may represent just the tip of the proverbial iceberg, as Tat is known to have more than 775 interactions with cellular proteins, and thus may interact with many more aspects of cell regulation (Self et al., 2004). On the other hand, the extent of MA-induced cellular damage is also not fully understood (reviewed in Salamanca et al., 2014). It should be noted that other factors such as gender, nutrition, immunological strength and genetic variation could significantly change the neurologic outcomes of HAND in MA abusers.

## Neurotoxicity by Methamphetamine Use and HIV-1 Infection: Potential Therapeutics

According to the CDC, as of 2010, the lifetime treatment cost of an HIV infected patient in the United States was an estimated $379,668. Moreover, the economical cost of substance abuse in the United States in terms of health care, job loss, corruption, imprisonment, and drug enforcement is more than $180 billion dollars a year (2008, NIDA). Unfortunately, the negative impact of HIV infection and drug abuse in our society is anticipated to deepen as the combined incidence of HAND, MA use, and HIV-1 infected MA abusers increases.

Under suppressive ART, HIV-1 patients are living longer, even as current evidence suggests their quality of life will degrade over time. Viral and human genetic variations, presence of latent cells and viral sanctuaries reduce the efficacy of ART (Aksenov et al., 2006). In addition, cofactors such as drug abuse significantly deepen HIV-induced neurological impairments.

As such, improving ART penetration across the BBB remains an important goal. The use of nano-ART (“cell-mediated medication delivery”; reviewed in Gendelman and Gelbard, 2014) or the use of intranasal delivery (reviewed in Hanson and Frey, 2007), have shown improved efficiency in that regard. The addition to ART of adjunctive therapies targeting the excitotoxicity, inflammatory or anti-oxidant systems is also being explored. Individually targeting Tat or MA is also another viable alternative. There are currently several ongoing clinical trials testing these approaches with MA-abusing HIV-1 patients to assess the inhibition of neurocognitive degradation or the decrease of MA use.

### Current Clinical Trials in HIV Infected Methamphetamine Users: Adjunctive Therapies

*Minocycline*, a tetracycline antibiotic currently used to treat bacterial infections, is a potentially exciting therapeutic agent to treat brain disorders including HAND (Chen et al., 2000; Du et al., 2001; Sanchez Mejia et al., 2001; Arvin et al., 2002; Van Den Bosch et al., 2002; Wu et al., 2002; Metz et al., 2004). Minocycline possesses several therapeutic indications including antioxidant, anti-inflammatory, anti-viral, and anti-addictive proprieties, and it crosses the BBB. Using cultured primary macrophages and lymphocytes *in vitro*, minocycline has been shown to prevent NMDA-induced microglial proliferation and activation (Tikka and Koistinaho, 2001; Tikka et al., 2001) as well as HIV and SIV replication (Zink et al., 2005). Tested *in vivo*, minocycline prevents inflammation and HIV replication in central and peripheral systems (Zink et al., 2005; Singh et al., 2014). At the behavioral level, mouse studies have demonstrated that minocycline decreases the rewarding proprieties of dextroamphetamine (a stereoisomer of amphetamine), MA and ethanol (Agrawal et al., 2011; Sofuoglu et al., 2011; Fujita et al., 2012). Moreover, minocycline improves cognitive deficits associated with exposure to phencyclidine (NMDA receptor antagonist) or MA (Fujita et al., 2008; Mizoguchi et al., 2008).

Minocycline has been tested in three clinical trials (NCT00855062; NCT01064752; NCT00361257) related to HIV-1-associated cognitive impairments. In the only results available to date, the phase II clinical trial with minocycline (NCT00361257) showed that it was safe and well-tolerated by individuals with HAND. However, no cognitive improvement was observed (Sacktor et al., 2011). Intake of minocycline only resulted in a decrease in lipid markers of oxidative stress (Sacktor et al., 2014), a disappointing result given the promise of preclinical studies.

*Ibudilast* (AV-411 or MN-166) is a non-specific phosphodiesterase inhibitor, used in Japan to treat ischemic stroke and bronchial asthma (Gibson et al., 2006). Ibudilast inhibits the activation of glial cells and the expression of macrophage migration inhibitor factors (Suzumura et al., 1999, 2003; Cho et al., 2010). It was shown to attenuate microglia activation by inhibiting the expression of pro-inflammatory cytokines, inhibiting markers of oxidative stress and increasing the secretion of anti-inflammatory mediators, which prevent neuronal death and white matter injury *in vivo* (Wakita et al., 2003; Mizuno et al., 2004).

Interestingly, ibudilast inhibits Tat mediated TNF-α expression by microglial cells (Kiebala et al., 2010; Kiebala and Maggirwar, 2011). It also inhibits Tat induced HIV-neuronal loss *in vitro* in the presence or absence of drugs such as morphine (Kiebala and Maggirwar, 2011; El-Hage et al., 2014). Moreover, ibudilast was reported to significantly inhibit HIV replication in microglia cells (El-Hage et al., 2014).

Ibudilast also effectively protects against the glial activation responsible for modulating MA-induced behaviors, such as hyperlocomotion activity and MA self-administration, as well as MA relapse caused by stress or re-exposure to MA (reviewed in Caputo et al., 2009; Beardsley et al., 2010; Snider et al., 2012, 2013). Ibudilast and an Ibudilast-analog that is ineffective at inhibiting phosphodiesterase, similarly suppressed MA self-administration in rats (Cho et al., 2010; Snider et al., 2013), raising questions over the mechanism though which ibudilast works.

Currently, there are 10 clinical trials testing ibudilast. Of these, NCT01217970 is a completed phase I clinical trial demonstrating the safety of the compound in combination with MA, whereas NCT01860807 is an ongoing phase II trial on HIV-1 patients.

### Other Adjunctive Drugs

Additional FDA approved compounds, currently effective for others brain disorders have been used in clinical trials for reducing MA use in HIV-1 patients such as aripiprazole, mirtazapin, and naltrexone.

*Aripiprazole*, which is a partial agonist at D2 receptors, is an antipsychotic used in the treatment of schizophrenia, bipolar disorder, depression and autism. It presented high toxicity when used in combination with ritonavir-boosted ART and cytochrome P450 inhibitor, reinforcing the need for caution when choosing the dosage and ART combination (Aung et al., 2010). In addition, depending on the dosage Aripiprazole has also been shown to increase MA-induced reward (Newton et al., 2008). In 2014, a phase II clinical trial (NCT00497055) was completed to test its efficacy to decrease the use of MA by HIV patients. However, the results are not yet available.

*Mirtazapine*, an antidepressant inhibitor of serotonin receptors, was reported to participate in the reduction of progressive multifocal leukoencephalopathy in one HIV-1 infected male (reviewed in Lasso and Ceron, 2012) and to reduce depression (Elliott and Roy-Byrne, 2000). It was used in a phase II trial in a cohort of HIV-1 men, which were actively using MA. In this study (NCT00497081), mirtazapine reduced MA usage and sexual risk.

*Naltrexone*, an opioid antagonist, was recently tested in a phase II trial (NCT01822132) in MA-using HIV patients to assess its ability to diminish MA-induced impulsive decision under observation with magnetic resonance imaging to determine functional correlate changes in brain. The results of this trial have not yet been made public.

Phase II clinical trials have been performed with this compound (NCT01449565; NCT00984360) for MA dependence on a cohort of men who have sex with men, due to the high percentage of this population in HIV infected MA abusers (NCT01449565), as well as on a cohort of individuals exhibiting the mutation A^118^ to G^118^ on the mu opioid receptor gene (NCT00984360). This mutation was shown to increase MA, alcohol and heroin addiction (Kuppuswamy et al., 1989; reviewed in Jeang et al., 1999; Peloponese et al., 2000; Chen et al., 2002; reviewed in Campbell and Loret, 2009). NCT00984360 was completed but the results are still not available, whereas, the NCT01449565 trial is still recruiting participants at the time of this review. In 2015, a study showed that naltrexone decreased the cue-induced craving and subjective responses to MA (Ray et al., 2015). This activity of the naltrexone had previously been observed with amphetamine and cocaine (Jayaram-Lindstrom et al., 2004, 2008a,b; Comer et al., 2013).

In parallel, several trials based on the *management* of HIV/MA are still ongoing^1^.

### Promising Candidates

BBB alteration, excitotoxicity, oxidative stress, neuro-inflammation and apoptosis all result from the disruption of different physiological pathways by Tat and/or MA. The review of Rumbaugh et al. (2008) describes different pathways involving Tat in HAND, which are mostly used in MA activities (reviewed in Rumbaugh et al., 2008). Therapeutically targeting cellular pathways requires using caution with dosages, as normally expressed human proteins are involved. Alternatively, one can physically target each toxin individually, which may be a less cytotoxic alternative.

#### Tat

Triptolide and didehydro-Cortistatin A (dCA), target the N-terminus and the basic domain of Tat respectively. These are currently the most promising Tat-targeting compounds, with an activity in the nanomolar concentration range.

***Triptolide***

Triptolide, is a diterpenoid epoxide isolated from *Tripterygium wilfordii*, which inhibits HIV-1 transcription by accelerating Tat degradation. Triptolide was described to have anti-inflammatory, immunosuppressive and anti-tumor properties (Wan and Chen, 2014). It is currently in a phase III clinical trial (NCT02219672), being tested in combination with ART on HIV-1 infected Chinese individuals for its ability to reduce the HIV-1 reservoir size.

***Didehydro-Cortistatin A***

Didehydro-Cortistatin A, is an analog of a natural steroidal alkaloid isolated from a marine sponge, *Corticium simplex*. It inhibits Tat-mediated transactivation of the HIV integrated provirus by binding specifically to the TAR-binding domain of Tat (basic region; Mousseau et al., 2012; reviewed in Mousseau et al., 2015). dCA promotes sustained silencing of the HIV promoter without viral rebound even in the absence of ART *in vitro*. dCA was also shown to inhibit Tat induced neuroinflammation of astrocytes *in vitro*, as well as Tat-induced cocaine potentiation *in vivo* (Mediouni et al., 2015).

For currently ongoing clinical trials for Tat vaccines^2^.

#### Methamphetamine

***Passive and active immunizations***

Passive and active immunizations against drugs of abuse are expected to slow down entry of the drug of abuse inside the brain thus preventing drug-associated behaviors. MA vaccines are still in preclinical development and promote low and variable level of antibodies production with moderate affinity for MA (Hauber et al., 1989; Mabrouk et al., 1991; Sabatier et al., 1991; Mousseau et al., 2012; Hambuchen et al., 2015). Research based on conjugate vaccine and adjuvant optimization should enhance the efficiency of these vaccines. Passive immunization with the human-mouse chimeric monoclonal antibody, mAb7F9, is already in more advanced stages of development (Hambuchen et al., 2015). Mab7F9 was reported to specifically bind to MA in the nanomolar range and mediate MA clearance (Laurenzana et al., 2014; Stevens et al., 2014). mAb7F9 attenuated MA induced hyper-locomotion even after irregular treatment and challenge with higher MA doses (Hambuchen et al., 2014; Laurenzana et al., 2014). It also partially reduced addiction-related behavior (Harris et al., 2015). Combination of MA hapten-conjugate vaccine to this antibody for 4 months reduced the presence of MA in the brain (Hambuchen et al., 2015). In 2014, this mAb was the first MA mAb to be used in a phase I clinical study in MA abusers (NCT01603147). The study was completed and confirmed the safety of the antibody. One potential caveat of this approach is that patients could be expected to increase their drug intake to overcome the effects of the antibody.

***Sigma receptors***

Methamphetamine and cocaine have been shown to directly interact with sigma receptors at physiological relevant concent-rations (Nguyen et al., 2005; reviewed in Robson et al., 2012). Sigma receptors have been shown to be involved in MA induced neurotoxicity through a variety of mechanisms such as neuroinflammation, hyperthermia, apoptosis, modulation of neurotransmitters and oxidative stress (reviewed in Kaushal and Matsumoto, 2011). In addition, sigma receptors show no homology to other human proteins, supporting the development of sigma receptor antagonists therapeutics to treat MA abuse (reviewed in Matsumoto, 2009). Several antagonists with nanomolar affinity have been developed. For instance, pretreatment with CM156 prevents hyperthermia and hyper-locomotor effects of MA as well as monoamine depletion in mice (Kaushal et al., 2011b), whereas AZ66 has been shown to prevent both MA-induced locomotor stimulation and the behavioral sensitization resulting from repeated MA administration in mice (Seminerio et al., 2012). Recently, SN79, which has been shown to have good “drugable” proprieties (Kaushal et al., 2011a), has mitigated MA-induced oxidative stress (Kaushal et al., 2014), apoptosis (Kaushal et al., 2011a), neuroinflammation (astrogliosis and microgliosis; Robson et al., 2013, 2014), hyperthermia (Kaushal et al., 2013, 2014), and neurotransmitters modulation (Kaushal et al., 2013). Moreover, SN79 also reverses cocaine-induced convulsions, locomotor activity and prevents sensitization to cocaine (Kaushal et al., 2011a). However, at higher doses, SN79 induces sedation and motor incoordination (Kaushal et al., 2011a). Currently, none of the sigma receptors antagonists are clinically tested in HIV-infected MA abusers or MA abusers cohorts.

#### Caveats

**FIGURE 4:**
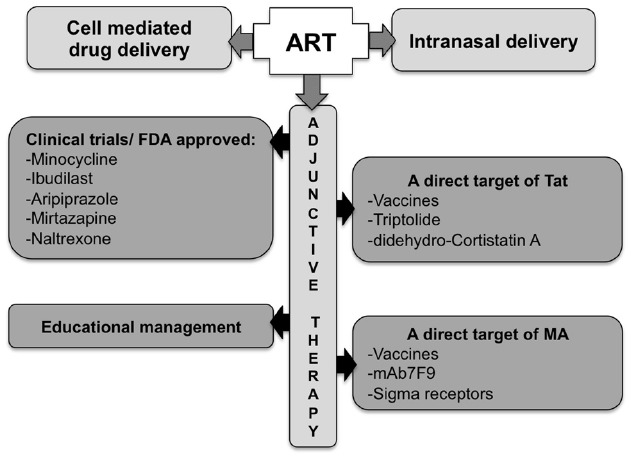
**Potential approaches for the treatment of Tat/methamphetamine toxicity.** The optimization of ART blood–brain barrier penetration may be achieved though a better route of administration, using carriers, or by including adjunctive therapy.

Finding the ideal therapy to treat HAND on the increasingly aging population of HIV-infected individuals, drug abusers in particular, is a significant challenge, especially with restricted animal models that fully reflect both HAND and drug abuse toxicity. The ideal therapy should block the pleiotropic activities of the toxic agent(s) without cell-associated toxicity, penetrate the brain or retain the toxic agent in the blood stream (e.g., antibodies), have low posology, present a high threshold for resistance development, and importantly, be inexpensive. Personalized therapeutic strategies may also be envisaged, given the human genetic variation (e.g., the mu opioid mutation A^118^ to G^118^, the HIV-1 co-receptor CCR5 Δ32 deletion), drug of abuse-induced epigenetic variations and HIV-1 genome variation (Kuppuswamy et al., 1989; reviewed in Jeang et al., 1999; Peloponese et al., 2000; Chen et al., 2002; Daily et al., 2006; Li et al., 2008; reviewed in Campbell and Loret, 2009). However, the cost of these strategies may be a hurdle. The combination of potent specific compounds (and/or immunizations) to ART could constitute a first step in the treatment of HAND (see Figure 4, for an overview of pathways and potential therapeutic tools).

## Conclusion

Although the biological effects of MA abuse and Tat protein have been extensively studied in the past, the result of their interaction is still not fully understood.

Methamphetamine abusers exhibit drug-related risk behaviors, such as needle sharing and unsafe sexual practices that increase the risk of acquiring an infectious disease. MA was shown to enhance HIV-1 replication, which may explain the rapid progression to AIDS of HIV infected MA users. On the other hand, Tat increases drug reward, which may partially explain the higher frequency of drug users in the HIV population.

Tat and MA act on homologous pathways, synergistically or additively, disturbing the same biological processes, including neurotransmitter pathways, inflammation, oxidative stress, and apoptosis. The outcome of this cross-interaction results in varying degrees of neurocognitive and locomotor disorders, that depend on patient complexity (reviewed in Valderas et al., 2009), HIV clade and drug use frequency. Compounds that target cellular pathways used for either Tat or MA-mediated cellular and behavioral activities, have the potential to reduce damage from both agents. Addition of these specific adjunctive compounds to ART is the most accepted treatment approach for HIV infected MA users. In addition, efforts on drug and HIV education and prevention, should be continuously provided.

### Conflict of Interest Statement

The authors declare that the research was conducted in the absence of any commercial or financial relationships that could be construed as a potential conflict of interest.

## Footnotes

1 https://clinicaltrials.gov/ct2/results?term=hiv+methamphetamine&Search+Search

2 https://clinicaltrials.gov/ct2/results?term=tat+vaccine&Search=Search
